# Boundaries can steer active Janus spheres

**DOI:** 10.1038/ncomms9999

**Published:** 2015-12-02

**Authors:** Sambeeta Das, Astha Garg, Andrew I. Campbell, Jonathan Howse, Ayusman Sen, Darrell Velegol, Ramin Golestanian, Stephen J. Ebbens

**Affiliations:** 1 Department of Chemistry, The Pennsylvania State University, University Park, Pennsylvania 16802, USA; 2 Department of Chemical Engineering, The Pennsylvania State University, University Park, Pennsylvania 16802, USA; 3 Department of Chemical & Biological Engineering, University of Sheffield, Mappin Street, Sheffield S1 3JD, UK; 4 Rudolf Peierls Centre for Theoretical Physics, University of Oxford, Oxford OX1 3NP, UK

## Abstract

The advent of autonomous self-propulsion has instigated research towards making colloidal machines that can deliver mechanical work in the form of transport, and other functions such as sensing and cleaning. While much progress has been made in the last 10 years on various mechanisms to generate self-propulsion, the ability to steer self-propelled colloidal devices has so far been much more limited. A critical barrier in increasing the impact of such motors is in directing their motion against the Brownian rotation, which randomizes particle orientations. In this context, here we report directed motion of a specific class of catalytic motors when moving in close proximity to solid surfaces. This is achieved through active quenching of their Brownian rotation by constraining it in a rotational well, caused not by equilibrium, but by hydrodynamic effects. We demonstrate how combining these geometric constraints can be utilized to steer these active colloids along arbitrary trajectories.

## Abstract (web summary)

Self-propelled colloidal particles can be potentially used to transport cargoes at the microscale, but it is challenging to prevent randomization of their motion by Brownian rotations. Here, Das *et al.* quench these rotations by solid walls, which guide in-plane swimming without the need for external fields.

## Graphical abstract

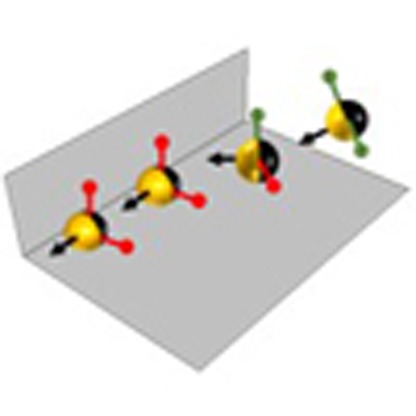

The ability to accurately steer self-propelled particles without the application of an external force field should have far reaching consequences. Such precise navigational control is essential for cargo transport^1^,^2^,^3^, repair and drug delivery within the body^4^, sensing^5^, environmental remediation^6^ and even micro-surgery targeting individual cells.

Self-propelled colloidal motors^7^,^8^, which transduce chemical energy into mechanical motion are an important class of active matter^9^,^10^,^11^. A critical challenge is in directing the motion of such active colloids, which include bi-metallic nanorods^12^, nanotubes^13^ and Janus spheres^14^. The major barrier is Brownian rotation, which randomizes particle orientations^15^, leading to long-time isotropic enhanced diffusion^16^,^17^. Current strategies for directing catalytic motors include external magnetic fields^18^, Earth's gravitational field^19^,^20^ and electrophoretic traps^21^. However, these strategies either lack autonomy^22^ (external fields and traps) or only constrain along one-dimension (gravity).

Here we introduce a new method of steering individual micromotors, using geometric boundaries, in a way that does not lead to ‘global' steering, as happens when external fields are applied. We demonstrate directed motion of a special class of catalytic motors—the spherical Janus colloid with half-coating of platinum with variable thickness—when moving in close proximity to solid surfaces through active quenching of their Brownian rotation, which leads to constrained in-plane swimming along the wall. Such prolonged directed transport is not dependent on any external fields or potentials and continues for length scales much larger than previously reported^23^. We find that a very specific set of criteria needs to be satisfied for this steering mechanism to work, and that it is the dynamic flow field—not the equilibrium interaction of the particle with the wall—that constrains the motion from enhanced 3D translational diffusion in the bulk to constrained 2D-enhanced diffusion at the walls. By combining geometric constraints, we demonstrate how it is possible to reduce the number of degrees of freedom for these autonomously moving catalytic colloids.

## Results

### Experimental characterization of the orientational quenching

Our system consists of Janus^24^ particles composed of platinum (Pt)-capped polystyrene (PS) colloids, which swim in solutions of hydrogen peroxide (H_2_O_2_) due to its catalytic decomposition into water and oxygen^14^. These Janus particles swim with the PS side forward, according to a self-electrophoretic mechanism, wherein ionic currents are set-up at the Pt end, between the poles and the equator^25^,^26^. We aim to study these colloids stochastic trajectories when they swim near an interface, so that we can characterize its effect on their active motion.

**Figure 1:**
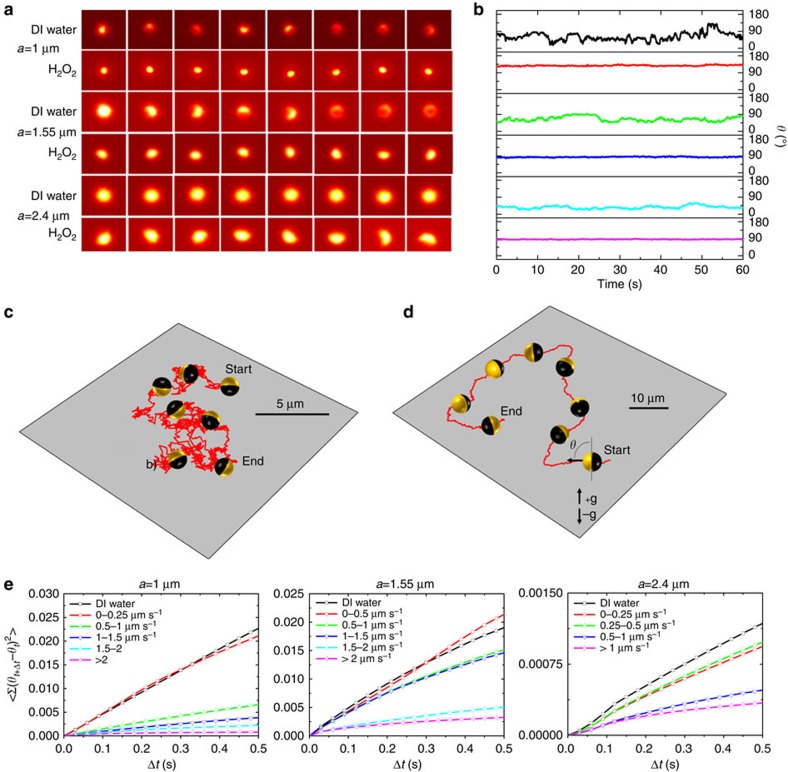
Brownian rotational quenching and alignment near a planar surface. (**a**) Selection of frames from fluorescent microscopy videos (15 × 15-μm field-of-view) for fluorescent platinum–polystyrene (PS) Janus spheres of varying radii, the PS side of the colloid appears bright (**a**), near to a planar interface in de-ionized (DI) water and in 10% aqueous H_2_O_2_ solutions. (**b**) Polar angle, *θ*(t) for typical Janus particles determined from fluorescent microscopy videos (note the *a*=2.4 μm particle in water shows strong gravitational alignment constraining *θ* close to 0°). (**c**,**d**) Schematic 3D orientation and experimental trajectories (45 s duration, red line) for Pt–PS Janus particle with radius *a*=1 μm in (**c**) DI water settled under gravity against a planar glass substrate, and (**d**) 10% H_2_O_2_ solution at either the top (+g) or bottom (−g) planar surface of a rectangular glass cuvette. (**e**) Polar mean square angular displacement (MSAD) as a function of time for three differently sized Janus spheres. In each graph, the black ‘Water' line represents the MSAD for Janus particle settled at a planar interface under gravity in water (*n*>20). The additional curves represent the MSAD for Janus particles with speeds in the defined ranges, at both the top (*n*>20) and bottom (*n*>20) planar surfaces of a rectangular cuvette.

The polar angle, *θ*, which probes how the polarity of a Janus particle is oriented with respect to the surface normal vector, and the corresponding in-plane orientation can be observed under fluorescence microscopy as ‘phases of the moon'^27^ (see Methods), Fig. 1. In the absence of H_2_O_2_, Janus particles sediment in a rectangular cuvette to the bottom wall and are observed to undergo Brownian translational and rotational diffusion about a cap-down equilibrium orientation dictated by gravity, due to the weight of the Pt (Fig. 1a,c). But in the presence of H_2_O_2_, the Janus particles undergo enhanced diffusion in the bulk and accumulate at both the top and bottom walls, according to the competition between gravitaxis^19^ and sedimentation (Supplementary Note 1). Strikingly, once close to the wall, for sufficiently high H_2_O_2_ concentrations, the swimmers maintain an orientation such that the half-moon shape is persistently visible (Fig. 1a,d). This suggests that the out-of-plane rotational diffusion is quenched so that the polar angle (defined in Fig. 1d) is *θ*≈90° (Fig. 1b and Supplementary Movies 1–3).

The relationship between propulsion speed and quenching becomes clear from mean square angular displacement (MSAD) curves for many Janus particles (*n*>25 at each condition) arranged by size and binned according to propulsion speeds (which was varied by changing fuel concentration, see Supplementary Fig. 1), along with a comparison to un-fuelled, purely Brownian Janus colloids (Fig. 1e). For particles undergoing unquenched Brownian rotation, we have 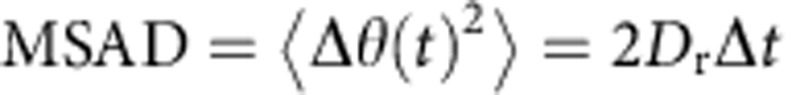. In water, a linear time dependence of the MSAD is observed at short time periods (MSAD trend towards an upper limit at longer times due to the periodic boundary conditions for the polar angle, 0°≤*θ*≤180°). However, as the magnitude of *v* increases, the MSAD becomes sub-diffusive after an initial period of linear time dependence at short timescales, and saturates at long times, to within our experimental resolution (Fig. 1e). The strength of the orientational quenching is manifestly increased as the propulsion velocity is increased.

### Steering the active colloid

**Figure 2:**
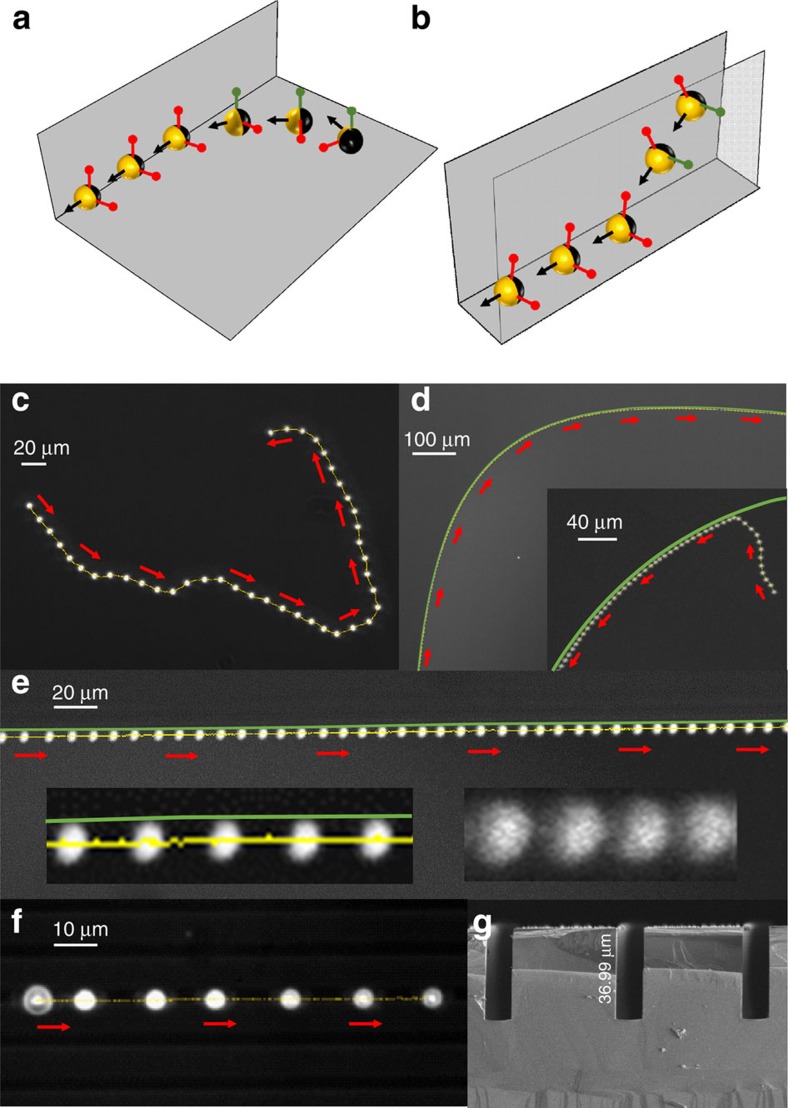
Particles moving along geometric boundaries, at speeds of up to 10 μm s^−1^. (**a**,**b**) schematics of Janus particles encountering multi-planar geometries. Red axis indicates forbidden rotations due to proximity to a plane; green axis indicates unquenched axis of rotation: (**a**) Janus particle encountering a planar edge while moving along a 2D surface, expected to result in Brownian rotational quenching about two orthogonal axes. (**b**) Janus particle confined within a square groove; parallel vertical walls confine the rotational diffusion about one axis; however, if the particle descends to the base of the groove, it is confined about two orthogonal axes. (**c**–**f**) Overlaid still frames from fluorescence microscopy videos with equal time gaps: yellow line shows complete trajectory, green line shows location of vertical cuvette walls and red arrows indication direction of motion: (**c**) *a*=1.55 μm Janus particle (10% H_2_O_2_) moving at the bottom of a rectangular glass cuvette a long way away from the edges. (**d**) *a*=1.55 μm Janus colloid (10% H_2_O_2_) moving along the curved edge of a glass cuvette—inset shows a colloid reaching the cuvette boundary. (**e**) *a*=2.4 μm Janus colloid (10% H_2_O_2_) moving along the straight edge of a glass cuvette—left hand inset shows a magnified region, right hand inset shows a ‘stuck' aligned agglomerate formed at the cuvette boundary. (**f**) *a*=2.4 μm Janus colloid (10% H_2_O_2_) moving within a rectangular section groove (width=8.75 μm). (**g**) S.E.M. image of a section of the rectangular grooves (widths 7–9 μm) used in **f**.

To further probe the role of geometry on the orientational quenching, we examine swimming of Janus particles near two orthogonal surfaces (Fig. 2a). We observe that a particle initially exhibiting 2D-enhanced diffusion at the surface (Fig. 2c) undergoes persistent linear motion when it reaches the vertical edge of the cuvette. Figure 2d,e shows examples of active colloids following curved and straight sections of the boundary for appreciable distances. The inset of Fig. 2d also shows the transition from 2D-enhanced diffusion to boundary steering occurring at the moment the colloid reaches the wall. In addition, the left hand inset for Fig. 2e (and Supplementary Movie 4) verifies that the colloid equator is aligned close to 90° as seen above for a single boundary. In fact, over many repeated experiments we observed that all the colloids investigated (which were of different sizes) were directed by the edge of the cuvette for length scales up to several centimetres. Colloidal motion continued around the entire macroscopic cuvette edge, only occasionally stopped by small blemishes (standard laboratory glassware that had not been precision engineered was used) or by encountering other stuck colloids, resulting in pile-ups of aligned colloids, Fig. 2e right hand inset. Due to the build-up of colloids following the edge, sometimes in different directions, collisions between moving colloids were also observed (Supplementary Movie 5). Collisions result in stable agglomerates, which continued to move along the boundary in a direction that qualitatively appeared to be determined by the relative propulsive velocities of the colliding components.

We also investigated an array of lithographically produced deep rectangular linear channels. Since the Janus particles now interact with three confining surfaces (two parallel walls and the bottom surface, as seen in Fig. 2b), geometric constraining should lead to a strictly linear motion of the Janus particles in the channels, with few or no ‘Brownian escapes'. Grooves with a variety of widths were investigated, and the colloids with radius *a*=2.4 μm were observed to be rotationally quenched and surface-aligned within channels with widths 7–9 μm. For example, Fig. 2f (and Supplementary Movie 6) depicts an active Janus colloid exhibiting persistent linear motion when confined within a 9-μm-wide channel, see Fig. 2g.

### Theoretical description of the catalytic colloid near a surface

Having determined the phenomenology of rotational quenching experimentally, we now set out to investigate this effect theoretically, and explore possible mechanisms that can account for these observations. The orientational dynamics of our active Janus particles could be affected by several mechanisms. These include equilibrium effects such as gravitational torque due to inhomogeneous weight distribution in the Pt cap and electrostatic interaction with the surface due to zeta potential difference between the two halves of the Janus particle. There are also non-equilibrium effects such as hydrodynamic coupling between the swimmer and the surface^28^,^29^,^30^, electro-osmotic effects due to the ionic activity of the active Janus colloid^25^,^31^ and electrostatic contributions due to additional difference in zeta potential between the two halves as a result of the non-equilibrium catalytic activity on the Pt cap. We take a pedagogical approach and construct an approximate theoretical description of the phenomenon, so that we can highlight the key physical features and the relative significance of the above effects.

There are several observations pointing to the fact that we observe a non-equilibrium effect which is a result of the propulsion mechanism of the particles. Higher concentrations of the fuel, H_2_O_2_, result in a greater fraction of time spent by the particle in the half-moon orientation. As expected, higher H_2_O_2_ concentrations also result in higher particle speeds both at the surface and in the bulk (Supplementary Fig. 1). Moreover, when the electrokinetic component of the propulsion mechanism is weakened by addition of 1 mM NaCl^25^, rotational quenching is also observed to decrease. We know that the gravitational torque is not responsible for the surface-aligned Brownian rotation quenching since no surface alignment is observed in the absence of H_2_O_2_, and the surface-aligned rotational quenching is apparently independent of the direction of gravity, as mentioned above.

### Electrostatic colloidal forces

**Figure 3:**
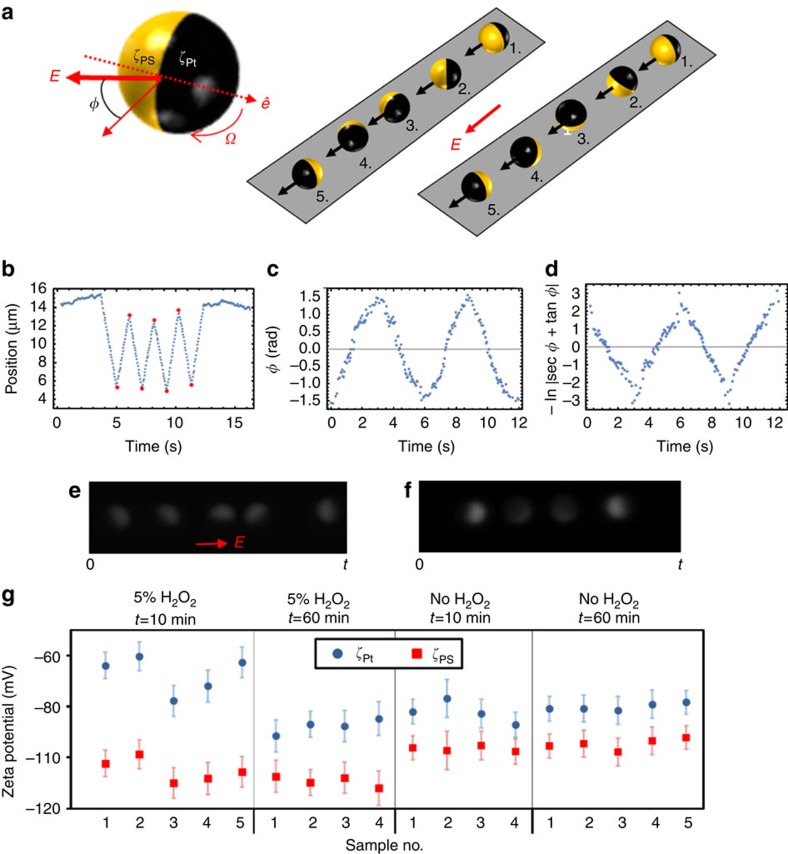
Electrophoretic behaviour for Janus colloids. (**a**) Schematic representation of the rotational electrophoresis experiment. Left hand side shows the relevant physical quantities, a Janus sphere with hemispheres with two different zeta potentials (*ζ*_Pt_ and *ζ*_PS_), giving a dipole vector 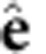. The dipole vector rotates in an electric field, and the right hand side 3D schematics depict the effect of switching the direction of *E*. Stage 1 represents the initial misaligned dipole and applied field orientation immediately after the E-field direction is switched, stages 2–5 show two possible rotations to re-align the dipole with the applied field: on the left hand side about an out-of-plane axis, with constant polar angle, *θ*, and on the right about an axis parallel to the plane where polar angle changes; at position 5 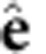 reaches the steady state. The black arrows show the direction of translational motion, which is always aligned with the applied field (see **b**). (**b**) Typical position versus time curves obtained by tracking a Janus particle (*a*=2.4 μm) above a glass interface with *E*_∞_=2.5 V cm^−1^ in 1 mM NaCl. **E**_∞_ pointed in the negative × direction first and then switched every second. The red circles are times when **E**_∞_ changed directions. (**c**) Typical *φ* versus time curve for rotation of a Janus colloid (2.5 V cm^−1^, 5% H_2_O_2_, 1 mM NaCl). We changed the direction of **E**_∞_ after the particle aligned with the applied field. (**d**) f(*φ*) versus *t* for **b** from equation (7) (see Methods). **e**,**f** show still frames from a fluorescence microscopy video for a Pt–PS Janus particle rotating about an out-of-plane axis (**e**) and about an axis parallel to the plane (**f**) from the point at which the applied E-field polarity was reversed to the depicted direction (red arrow). (**g**) Measured zeta potentials for both Pt (blue markers) and PS (red markers) at two time points following sample preparation, each with and without hydrogen peroxide. Error bars represent the zeta potential s.d. determined from at least four field reversals.

If the surface potentials of the particles were to change significantly in the presence of the reaction, electrostatic colloidal forces could contribute to the rotational quenching. To estimate the contribution of the electrostatics with and without catalytic activity, we measured the zeta potential of each end (Pt and PS) of the Janus particle by combining both translational and rotational electrophoresis experiments in 1 mM NaCl, Fig. 3a. In carrying out these measurements, the presence of bubbles due to the reaction at Pt end produces two challenges, which we addressed: (1) increased resistance to electric current, since the bubbles occupy cross-section where the current could flow, and (2) complex convective flows around bubbles. We designed a disposable electrophoresis set-up (Methods), applied a constant current across it, and worked in cross-sections of the cell where we observed no bubbles, to get a constant electric field in the solution phase. The translational velocities of Janus particles were measured with respect to velocity of tracers that had a known zeta potential, to account for any convective flows, including electro-osmotic flow near the cell wall. The transport of the Janus particles and tracers were always found to be smoothly linear.

As shown in Fig. 3b, we see typical translational electrophoresis motion of a Janus particle (*a*=2.4 μm) in a 2.5 V cm^−1^ E-field in the direction of the E-field. The average zeta potential (*ζ*_J_) was interpreted using the Smoluchowski equation. Averaged over many particles, we found an average of *ζ*_J_=−87±5 mV (in 1 mM NaCl) with no fuel present, and *ζ*_J_=−98±6 mV with 5% H_2_O_2_ present (Fig. 3g, and Supplementary Table 1).

We next measured the difference between the Pt and PS sides of the particle. Owing to unequal zeta potentials at each end, the particle undergoes rotational electrophoresis to align the zeta potential dipole with **E**_∞_ (Fig. 3c–f); see Methods^32^. The contribution of complex convective flows to rotation is negligible in the presence of electric field as can be inferred from the final steady positions of the dipole (Fig. 3c). We tracked particle rotations under fluorescence microscopy and used the measured angular velocity to obtain the zeta potentials for each case (Fig. 3d,g). The zeta potential of the PS end does not change appreciably upon the addition of fuel. We also find that the zeta potential of the Pt end stabilizes to a constant value, after about an hour in H_2_O_2_. While we observe some degree of sample-to-sample variability, based on the above measurements, we can largely conclude that the values of the zeta potentials of the two ends change by a small amount in relative terms due to the non-equilibrium catalytic activity. Hence, we can safely rule out electrostatic interactions as the main cause of rotational quenching.

### Phenomenology

**Figure 4:**
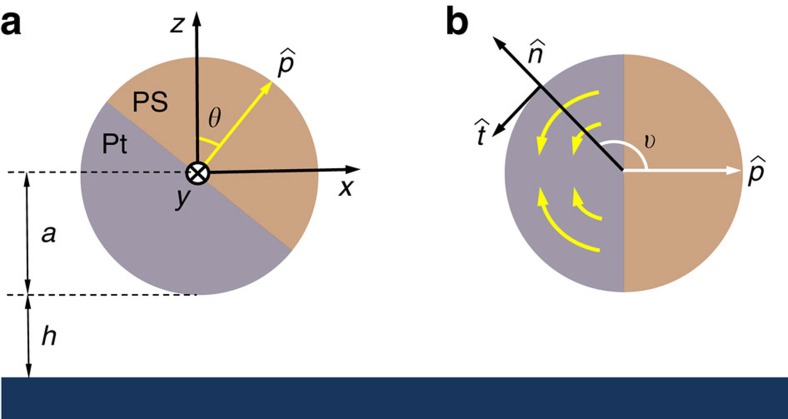
Schematics of the colloidal swimmer near a substrate. (**a**) Geometry of the system.. (**b**) The surface slip velocity flow field. 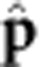 indicates the direction of propulsion.

Driven motion of a sphere through viscous fluid near a solid substrate that imposes no-slip boundary condition introduces coupling between translation and rotation. We can get an intuitive idea of why this coupling comes about by thinking of a singular limit of the problem, where a solid sphere in no-slip contact with a substrate is pulled by an external force; the sphere rolls along the surface. In the case of a sphere of radius *a* that is moving above a substrate at the (closest) distance of *h* (see Fig. 4), the asymmetric shear stresses around the sphere will lead to rotation in addition to translation when the sphere is pulled through by an external force parallel to the wall. While this is a comparatively small effect when *h*>>*a*, for small gap sizes *h*≤*a* the resulting translation and rotation are of the same order of magnitude and the coupling cannot be ignored (see Supplementary Table 2).

For simplicity, we consider a reduced 2D version of the problem in which both translation and rotation of the sphere are restricted to the *xz*-plane, where the *x*-axis corresponds to the direction of the translational motion and the *z*-axis is perpendicular to the surface of the substrate (see Fig. 4a). The orientation of the sphere is described by the angle *θ* that the director of the Janus particle makes with the *z*-axis.

The stochastic motion of the colloid, which is under the influence of surface interactions with a potential *U*(z, *θ*) as well as gravity, can be described via the following Langevin equations


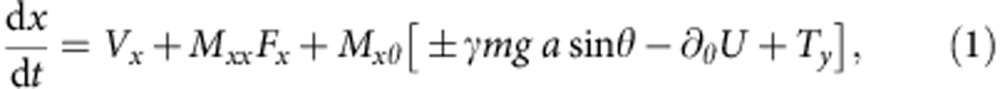



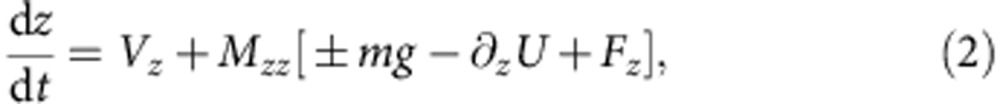



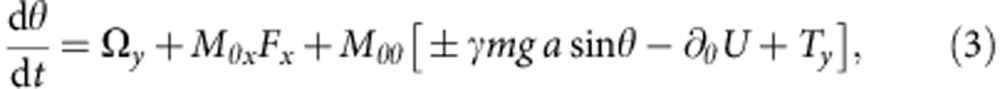


where *V*_*x*_(*z*, *θ*) and *V*_*z*_(*z*, *θ*) are the corresponding components of the spontaneous translational velocity of the particle, Ω_*y*_(*z*, *θ*) is the corresponding spontaneous angular velocity and *M*_*αβ*_(*z*, *θ*) are the relevant mobility coefficients. Note that *M*_*xθ*_=*M*_*θx*_ (ref. 33). The expression for gravitational torque contains *γ* that is a numerical constant of order unity that depends on the details of the weight distribution across the Janus particle (caused by the metallic cap). The choice of sign depends on the direction of gravity. Finally, *F*_*x*_, *F*_*z*_ and *T*_*y*_ are noise terms.

The Langevin formulation can be used to construct an alternative description via the probability distribution 
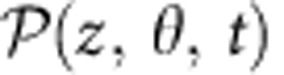
 that satisfies the Fokker–Planck equation 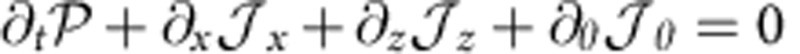 where the fluxes are defined as


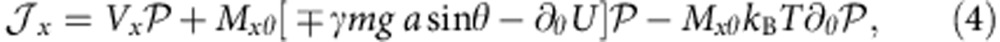



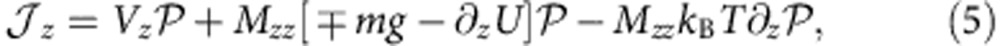



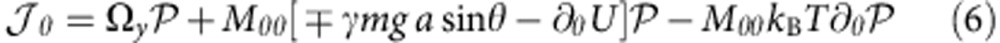


The *x* dependence is eliminated due to translational symmetry.

We take a pedagogical approach, and aim to construct an approximate solution to the above equation, so that we can highlight the key physical features. In stationary state, *V*_*z*_ and ∂_*z*_*U* will have relatively weak dependence on *θ*. We can obtain a stationary height *z*_s_=*a*+*h* by setting 
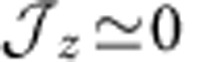
, from the balance between the interaction with the surface, gravity and the average vertical component of the self-propulsion, possibly determined by the gravitational angular deflection due to the bottom-heaviness of the Janus particle. In the vicinity of the stationary height, we can find an approximate expression for the *θ* dependence of the stationary distribution by setting 
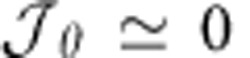
:


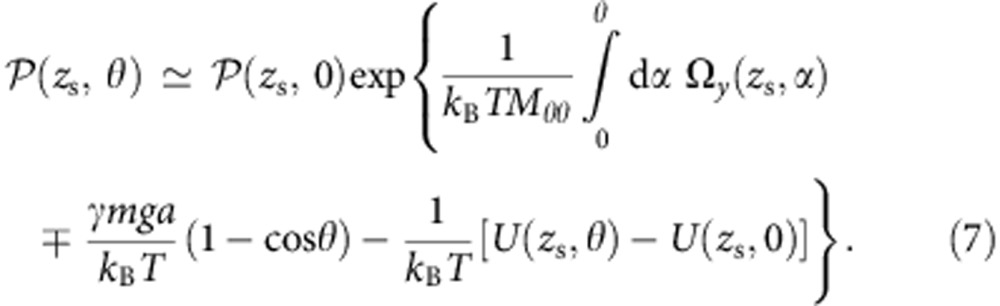


In the above equation, 
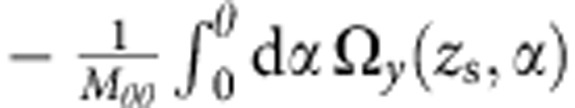
 acts as an effective potential that represents the non-equilibrium activity of the colloid due to the catalysis.

We know from the experiments that the gravitational term has a small effect and can be ignored for most colloids (except for the largest radius). The equilibrium surface interaction potential *U*(*z*_s_, *θ*) has contributions from different sources such as the electrostatic interactions due to the zeta potential difference across the Janus particle. The experiment tells us that the strong orientation quenching only exists when there is catalytic activity, which suggests that this term too can be neglected as compared with the non-equilibrium component. Therefore, we can write a simplified form for our approximate expression:


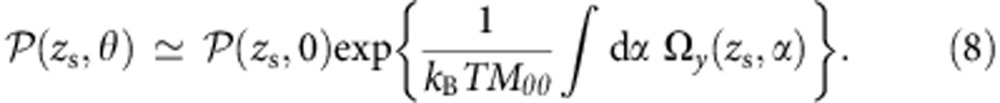


The experimental observations are consistent with the phenomenological Hookean form of


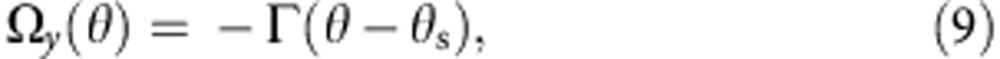


for the angular velocity, where 
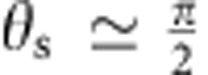
 gives the stationary orientation and Γ>0 acts as an effective (restoring) elastic constant. Based on the observation that the quenching effect increases with increasing fuel concentration, as well as dimensional grounds, the parameter Γ depends on the particle velocity and the radius of the sphere as


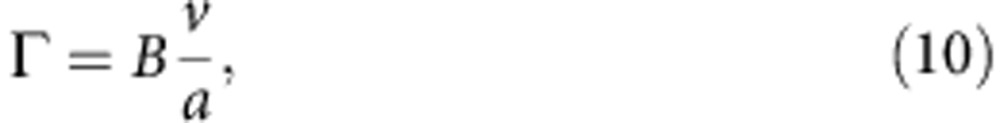


with the dimensionless pre-factor *B* depending solely on the material and geometric parameters, such as zeta potential, height and radius (see below).

The gravitational potential and the equilibrium surface interaction potential contributions in equation (7) will shift the equilibrium angle *θ*_s_ by a small amount. Putting equation (9) back in equation (8), we obtain


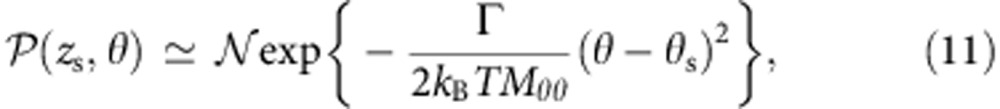


where 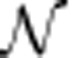
is a normalization constant.

We now go back to the Langevin equation for angular fluctuations equation (3) and use it to calculate the MSAD. The correlations of the noise terms defined via the generalized friction coefficients are as follows


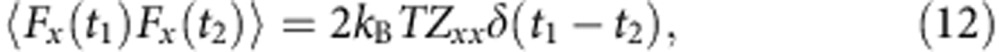



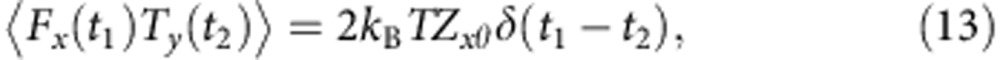



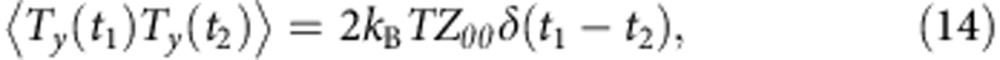


can be related to the mobility coefficients through the following identity


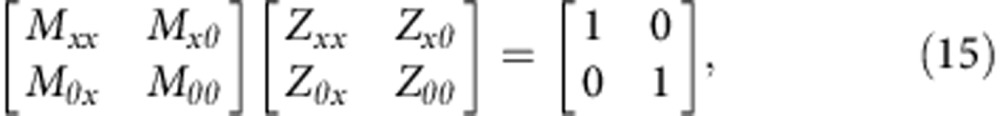


due to the fluctuation–dissipation theorem. By solving the Langevin equation, we find the MSAD as


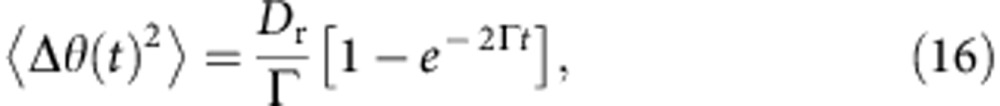


where the Einstein–Stokes relation *D*_r_=*k*_B_*TM*_*θθ*_ is used for the rotational diffusion coefficient to ensure the short time asymptotic behaviour of the above expression has the appropriate slope as expected for rotational Brownian motion.

**Figure 5:**
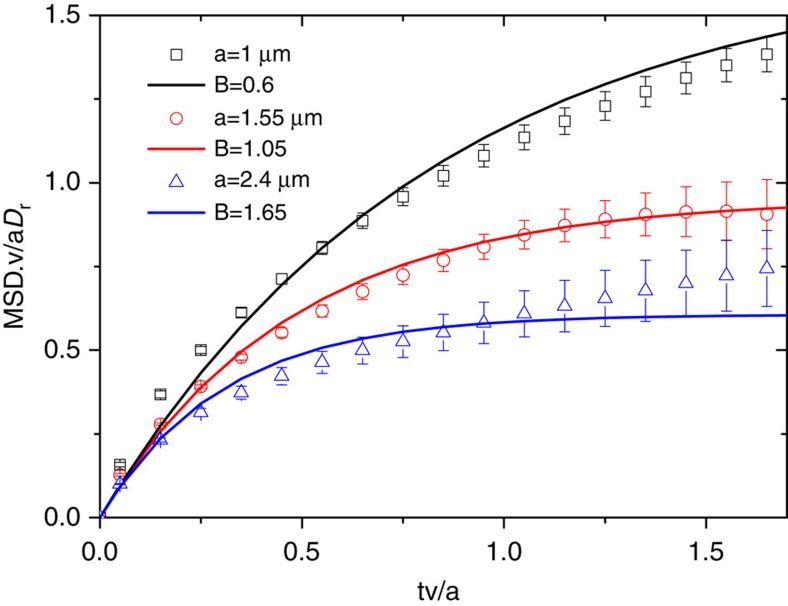
MSAD data re-scaled to allow comparison with theory. Lines are fits to equation (16) with estimated values for *B* (see equation (10)). Error bars represent the s.d. for each MSAD value.

Values of *B* can be obtained experimentally by plotting the averaged and re-scaled rotational MSAD data as a function of time (Fig. 5). Data collapsed for a large number of individual trajectories at each particle size gives values of *B* of order unity.

### Mechanism

The parameter Γ that quantifies the degree of orientational quenching exhibits a dependence on the non-equilibrium catalytic activity that is controlled by the net propulsion velocity. We now examine different mechanisms that could describe the observed rotational diffusion quenching, and show that they lead to the behaviour described by equations (10) and (16), and estimate their corresponding contributions to *B*.

When a sphere is pulled by a mechanical force along the horizontal axes, the presence of the surface induces a coupling that makes it roll (that is, Ω_*y*_>0). Phoretic transport of colloids with uniform surface properties has been shown to exhibit a peculiar anti-rolling behaviour (that is, Ω_*y*_<0)^34^. In self-phoretic propulsion^9^, a force-free and torque-free Janus colloid takes advantage of gradients that are generated due to the asymmetric activity across its surface to generate phoretic motion^35^. When a Janus particle self-propels in the vicinity of a surface, the coupling could lead to a mixture of rolling and anti-rolling tendencies, depending on the geometry and the type of activity^36^,^37^. This provides a promising starting point as it might be possible to find a specific combination that could lead to a fixed point as described by equation (9) and find the criterion for its stability, that is, Γ>0. The effect combines contributions from the phoretic and hydrodynamic interactions with the surface^36^. The problem cannot be solved exactly in 3D, and a purely computational approach might not be particularly illuminating with regard to the exact mechanism that can explain the observed quenching. We use an alternative approach and estimate the contributions of various possible mechanisms using asymptotic approximations and scaling arguments.

The hydrodynamic flow field generated by colloidal particles that are transported via phoretic mechanisms are most commonly described by sources and sinks on their surfaces, in the form of a slip velocity **v**^s^ rather than prescribed forces^38^. For such a system, we can employ a far-field approximation, which has been shown to account for the hydrodynamic effect with high accuracy even in the vicinity of the surface^28^,^29^,^30^, to show that Ω_*y*_ behaves as equation (9) with


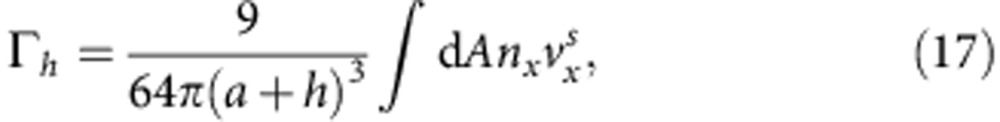


to the leading order (see Fig. 4b). equation (17) suggests that to have stable orientational quenching (that is, Γ>0) for spherical self-phoretic swimmers, two very specific criteria need to be met. The first criterion comes from symmetry: any surface slip velocity profile that is fore-aft symmetric will result in Γ=0. This implies, for example, that the diffusiophoretic component of the surface slip velocity that is fore-aft symmetric cannot lead to the observed quenching of orientation. This type of surface slip velocity profile is known to lead to hydrodynamic flow field that decays as 1/*r*^2^, which leads to a stronger hydrodynamic coupling than the fore-aft symmetric contribution that decays as 1/*r*^3^ representing a symmetric source-dipole^39^. The second criterion involves the direction of swimming versus the catalytic coating; Γ>0 only when the Janus particle swims away from the catalytic patch. The main motility mechanism of our Pt-coated Janus particles involves proton current loops that emanate from the vicinity of the equator and end near the pole^25^, hence satisfying both of these criteria by serendipity. For the sake of presentation, we use a representative velocity profile as 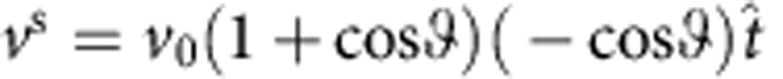 for 
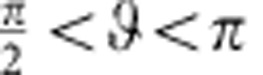
 (see Fig. 4b), which should provide a very good approximation to the exact velocity profile, and find


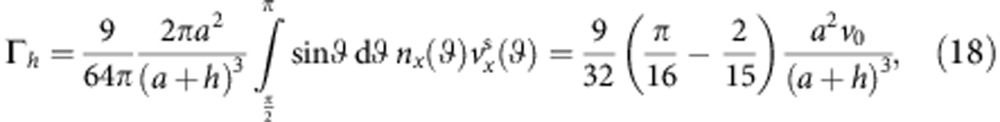


which is positive. In equation (18), *v*_0_ is a characteristic velocity scale and 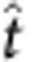 is the unit tangent vector along the direction of increasing 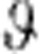. To relate *v*_0_ to the net propulsion velocity *v*, we can use the following approximate expression


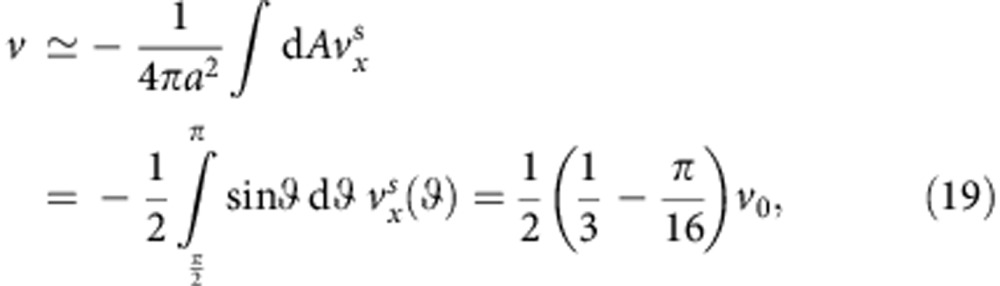


which should give us a reasonable estimate. Combining the above results, we find 
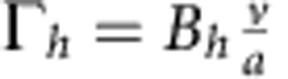
, where 
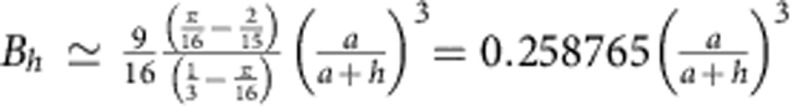
. This result is in the expected form of equation (10). We expect the pre-factor 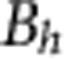 to be stronger than the above estimate when the colloid is in close proximity of the surface (see Supplementary Table 2). This is consistent with the experimental observations that give out fitted values for *B* that are of order unity. Recent observations on the motion of catalytic Janus spheres inside the matrix of a colloidal crystal reported by Poon and co-workers^23^ are consistent with the existence of such a strong hydrodynamic coupling arising from a 1/*r*^2^ decay due to the breakdown of the fore-aft symmetry in the surface slip velocity profile, as described above.

**Figure 6:**
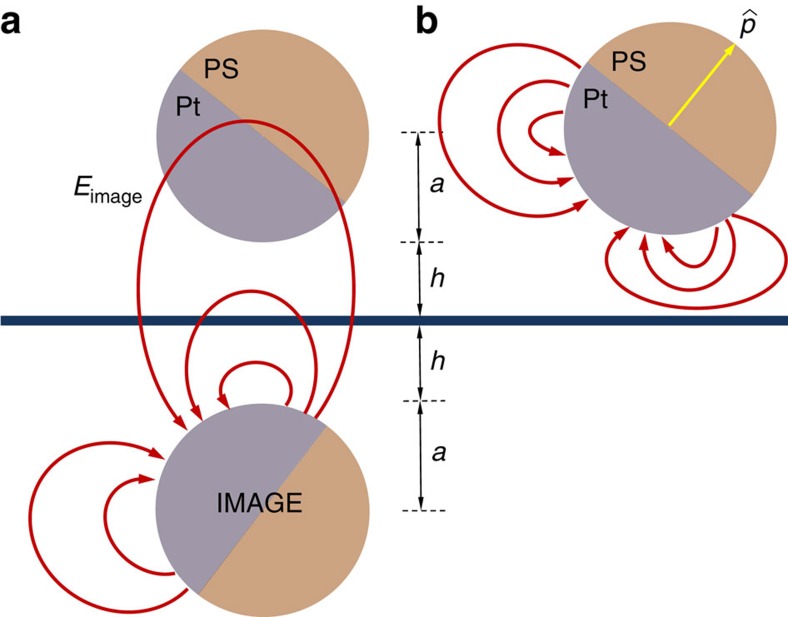
Schematics of the colloidal swimmer near a substrate. Showing the field lines created by the image distribution and *E*_image_ at the location of the Janus sphere (**a**) and the real electric field lines (**b**).

We know that the self-propulsion in our Janus particles is predominantly controlled by electrokinetic effects^25^. Therefore, it is natural to expect to have a significant electric field that results from the surface proximity of the proton currents caused by the catalytic activity of the Janus particle; the so-called ‘image field' (see Fig. 6). Due to the difference in the zeta potentials between the two halves of the Janus particle, the image field could contribute to the aligning tendency of the Janus particle via an electrophoretic contribution. In the bulk, we can relate the propulsion velocity to the self-generated electric field (caused by proton currents) using the Smoluchowski equation, as 
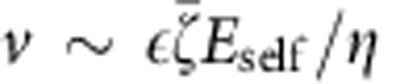
, where 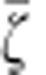 is the average zeta potential of the two halves of the Janus particle. The presence of the surface will modify the proton currents and the resulting electric field, described by the image distribution. The electric field caused by the image at the location of the Janus particle, will create an angular velocity as given in equation (9) with the coupling 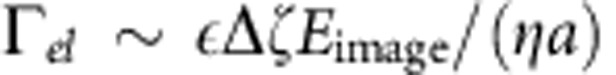, where Δ*ζ* is the difference between the zeta potentials of the two halves of the Janus particle. Considering the geometry as can be seen from Fig. 6, we can relate the strengths of the electric fields as 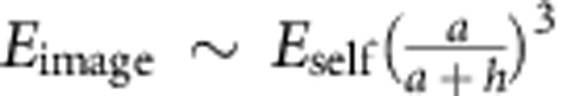 due to their dipolar nature. This can be combined with the above results to give the estimate 
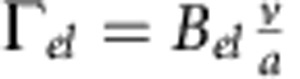
, where 
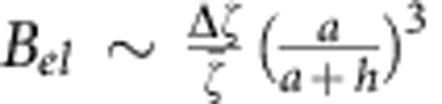
. Therefore, we conclude that our estimate of the electrophoretic contribution Γ_*el*_ is sub-dominant (though not negligible), as it is smaller than our estimate of the hydrodynamic contribution Γ_h_ by a factor of 
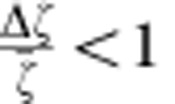
. Note that this statement uses the parametric form of the expressions we have obtained using scaling arguments, and thus the numerical prefactors in both estimates, which are likely to be comparable, are ignored for this comparison.

We can also estimate the contribution from electro-osmotic flows generated by the electric field in the vicinity of the wall. Unlike the case of electro-osmosis under externally applied electric field, the electric field generated by the proton fluxes is localized in the vicinity of the colloid only. The magnitude of this electric field near the wall will be given by *E*_image_ up to a numerical pre-factor (see Fig. 6), and it will give rise to a localized surface-bound electro-osmotic flow of 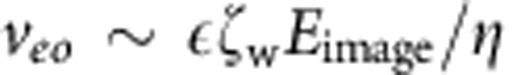. This expression can be rewritten in terms of the propulsion velocity as 
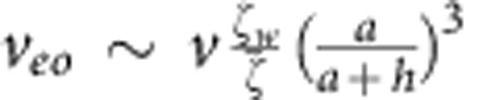
, which can then be fed back to our calculation of Γ in equation (9) as an additional contribution to the surface slip velocity, which yields 
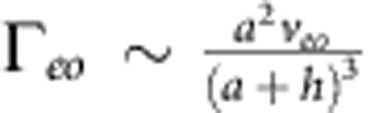
 and 
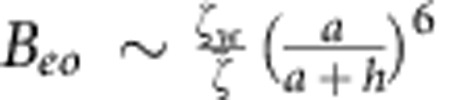
. Therefore, we conclude that our estimate of the electrophoretic contribution Γ_*eo*_ is smaller than our estimate of the hydrodynamic contribution Γ_*h*_ by a factor of 
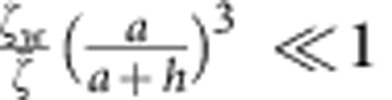
 within our approximation scheme.

Finally, we estimate the contribution from self-diffusiophoresis. In the style of the above estimates, diffusiophoresis can be represented by a slip velocity of the form 
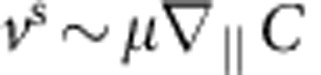
, where *μ* is a characteristic diffusiophoretic mobility and ∇_||_*C* gives the tangential gradient of a relevant concentration field. In a realistic description of the reaction scheme, we will need to use an algebraic sum of such terms representing each constituent of the reaction^38^. For half-coated Janus configuration, an angular velocity is proportional to the difference between the mobilities on the two halves Δ*μ* and the relevant component of ∇_||_*C* will be due to the image of the source/sink with respect to the impenetrable wall, which is a factor of 
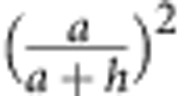
 smaller than the local ∇_||_*C* that is responsible to self-diffusiophoretic propulsion^37^. Using this scale for the slip velocity in equation (17) gives the self-diffusiophoretic contribution as 
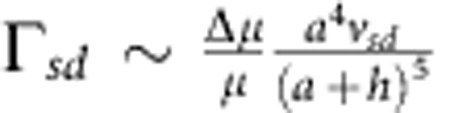
 and 
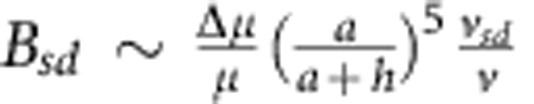
. Therefore, we conclude that our estimate of the electrophoretic contribution Γ_*sd*_ is smaller than our estimate of the hydrodynamic contribution Γ_*h*_ by a factor of 
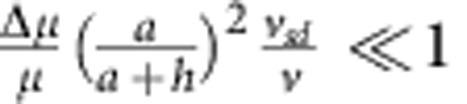
 within our scaling argument, since we know^25^ that 
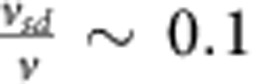
 and 
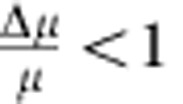
.

The above perturbative analysis of the three effects shows that the unique quenching behaviour is a result of the hydrodynamic interaction of a force-free, self-electrophoretically propelled, particle with the wall and the broken symmetry of the surface flow field. It is the asymmetric slip velocity profile that differentiates these metal–insulator particles from other active particles propelled by self-electrophoresis such as bi-metallic Janus particles, as well as self-diffusiophoretic Janus spheres, where the slip velocity is symmetric and quenching by this mechanism is not expected. Moreover, if the surface interactions are modified such that the Janus sphere swims with its Pt cap forward, then this mechanism cannot lead to stable orientational quenching. These spherical Janus swimmers are a special class, in their ability to be steered by boundaries (see Supplementary Note 2). It is hoped that in this context rotational confinement will be investigated in the future in a wider range of active colloid systems. For example, a bi-metallic Janus particle^40^ has been reported, and fluorescent labelling could allow the above prediction to be tested.

Theoretically, we can estimate the mean residence time of the active colloids by roughly defining escape as jumping over the relevant barrier in the effective non-equilibrium potential. Although we have only calculated such a potential for small angular deviations, we can assume that the full potential will be a combination of trigonometric functions with the quadratic small angle limiting form. This suggests that the overall strength of the potential will still be controlled by Γ. Then we can roughly estimate a mean residence time in the form of 
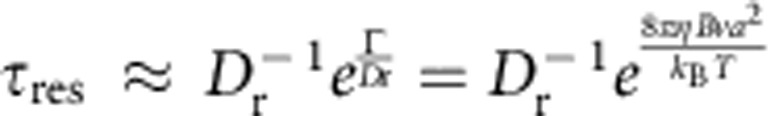
. Using the experimentally determined size dependence of the velocity 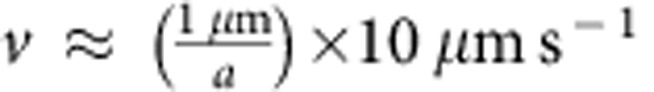 (ref. 41), we find that the residence time effectively depends on the exponential of the size (a weak dependency) and is significantly longer than the rotational diffusion time for *a*>50 nm.

## Discussion

Here we show that geometric constraints can help steer our Janus particles, by lowering the effective dimensionality of the space on their trajectories. One surface constrains the motion in 2D, and two perpendicular surfaces constrain it to 1D. This can be further exploited to create more elaborate constraints. For example, we expect a corner with three perpendicular surfaces to act as a trap and fully constrain the motion of our active colloids.

The ability to steer Janus motor particles uni-directionally along complicated trajectories by simply following an edge or groove opens the door for many transport and separation tasks such as directed cargo delivery, motility-based sorting and flow-free microfluidics. Feature-directed steering combined with single-particle^42^ and collective many-body^43^ chemotactic response could provide an ideal toolkit for designing novel strategies to be employed in oil or mineral exploration tasks^44^. Also as evident from Fig. 2e this is a useful method for reversible bottom-up assembly of active colloids and gives us a unique advantage in controlling the orientation of the assembled active Janus particles. One further possibility opened up by understanding the mechanism is that in addition to ‘geometric railroad tracks', we could take advantage of chemical patterning of the surface to achieve a higher degree of control when we guide particles along pre-determined paths. As a final comment, nature frequently produces motion at micron scales using moving parts to displace fluids, and in these systems hydrodynamic interactions between moving cellular structures and interfaces also leads to confinement at interfaces^28^,^45^. Here we have shown that orientation confinement for phoretic synthetic active colloids with no moving parts can result in phenomenologically equivalent behaviour.

## Methods

### Janus particle preparation

Catalytic Janus spheres were prepared by spin coating a 0.1 wt % dispersion of fluorescent PS microspheres (Themoscientific−radius=1, 1.55 and 2.4 μm) from ethanol onto freshly cleaned glass microscope slides. A 10-nm-thick layer of Pt (>99.9%, Sigma-Aldrich) was then evaporated onto one side of the microspheres under vacuum in a Moorfield (U.K.) Minilab 80 e-beam evaporator. Low volume fraction solutions of colloids in pure water (resistivity>15 MΩ) or the specified aqueous concentration of H_2_O_2_ (>99.9%, Sigma-Aldrich) were prepared by removing the colloids from the glass slides by either ultra-sonication or physical transfer onto lens tissue and re-suspension.

### Microscopy and image analysis

The active colloids were observed using the fluorescence mode of a Nikon Eclipse LV100 microscope with illumination through blue excitation band of a Nikon B2A filter cube. Videos were captured using an Andor Neo camera at a frame rate of 33 Hz. Custom software developed using LabView vision identified the *x*,*y* coordinate for the centre of each bright particle throughout each video, thus generating trajectory data. Such trajectories can be quantified using mean square displacement versus time analysis to determine propulsion velocity as previously described^16^.

The software also determined the average pixel intensity for the central region of each tracked particle as a function of time. For a given particle size, illumination and camera settings were fixed, allowing quantitative comparison between these intensity values. To convert these relative fluorescence emission values to polar angles, it was necessary to establish the intensities that corresponded to cap-down (*θ*=0° maximum brightness) and cap-up (*θ*=180° minimum brightness) orientations. For *a*=1- and *a*=1.55-μm particles a self-calibration approach was used, where long duration intensity versus time plots were obtained for a number of particles in water, and the average maximum and minimum intensities were assigned to *θ*=0° and *θ*=180° polar angles. The intensity between these limits was assumed to vary sinusoidally to complete the conversion^27^. However, for *a*=2.4-μm Pt–PS Janus particles the gravitational torque makes the probability of rotation to a cap-up configuration too low to observe within a reasonable time frame. To overcome this, *a*=2.4 μm colloids were ‘frozen' within a transparent gellan gum, allowing the now solid sample to be inverted. The minimum intensity found from observing many of these inverted particles was used as the *θ*=180° limit, subsequently allowing conversion to polar angles as before. All presented polar angle data were obtained in this way; however, the data collapse shown in Fig. 5 also constrained the short term behaviour to match the theoretical value for the rotational diffusion coefficient to further correct for particle-to-particle variations in fluorescence intensity.

### Electrophoresis/rotational electrophoresis

Standard dynamic light scattering (DLS) equipment is not designed to handle either the material heterogeneity of metal–insulator Janus particles or bubbling at the electrodes in the presence of H_2_O_2_. Measurement of *ζ*_Pt_ and *ζ*_PS_ using separate homogeneously coated Pt and PS particles is possible, but a constant thickness Pt surface does not undergo the same reaction as a Janus particle with an inhomogeneous coating on the Pt half^25^. Consequently, to measure the average colloid zeta potential, *ζ*_J_, we carried out translational electrophoresis in a homemade closed capillary set-up using the following protocol.

The Pt-coated glass slide covered in Janus colloids was submerged in 25 ml de-ionized (DI) water in a petri dish and sonicated for 30 min. The resulting dispersion was concentrated to 3 ml by centrifugation at 1,000 *g* in a Sorvall Biofuge Primo centrifuge. About 50 μl of the concentrated Janus solution was diluted to 600 μl using 1 mM NaCl solution containing 0.003% by volume of sPSL tracers. For samples with H_2_O_2_, equal volumes of concentrated Janus particle solution and 30% H_2_O_2_ were sonicated for 5 min and left for another 20 min to activate the Pt surface. Then they were diluted to 5% H_2_O_2_.

The solution for analysis was fed into a cleaned glass capillary (0.9-mm^2^ cross-section, 50-mm long and 0.18-mm wall thickness from Vitrocom, RCA-I cleaned) and placed on a glass slide making sure there are no bubbles or air gaps. About 1.5-cm long piece of gold wire (0.5 mm, 99.99% purity Alfa Aesar, cleaned using water and ethanol) was inserted at each end and the capillary was sealed off using wax and a ultraviolet-curing adhesive. The capillary set-up was mounted on the motorized stage of a Nikon TE 300 inverted microscope, so that the capillary surface faced the × 20 or × 40 objective. The gold wires were connected to electrodes from a Keithley 2,410 source meter, which was operated in the constant current mode at 3 μA, resulting in an electric field of 2.5 V cm^−1^.

To validate our technique, we measured the zeta potential of sulfated PS latex (sPSL) tracer particles using our set-up and compared it with the measurements from the DLS-based Malvern Zetasizer. To do so, we measured the translational velocity of particles in eight different planes, and used Bowen's equation^46^ to fit for *ζ*_spsl_ and *ζ*_wall_ (Supplementary Fig. 2a). Zeta potential of sPSL particle (*ζ*_spsl_),was measured using DLS (Malvern Nano ZS Zeta Sizer), as well as using our set-up, which served to verify that our set-up did not introduce impurities to the system (Supplementary Fig. 2b). Limited available volume fraction and fast settling did not permit a similar experiment on Janus particles to measure zeta potential of the Janus particle (*ζ*_J_) directly.

Two problems arise due to the formation of oxygen bubbles when conducting experiments in the presence of H_2_O_2_ fuel. First, there is increased resistance to current flow, but we managed to keep the electric field constant by applying a constant current through the capillary. Second, bubbles lead to convective flows in the capillary, which we account for by measuring translational speeds relative to tracer particles with known zeta potentials. These considerations resulted in the following methodology:

1. We measured the zeta potential (*ζ*) of sPSL, in 1 mM NaCl, both with and without H_2_O_2_. We wanted to test whether the H_2_O_2_ made a difference on the zeta of only the sPSL, with no Janus particles present. The data showed that the zeta potential of sPSL does not change in the presence of H_2_O_2_, to within the statistical uncertainty of about 5%.
2. We then followed our Janus particles, using the sPSL as tracers. We did this away from any visible bubbles. The particles had a smoothly linear motion, with no visible effect of any complex flow fields that might have resulted from bubbles.
3. Knowing *ζ*_SPSL_, we were able to assess *ζ*_Janus_, including uncertainties. Importantly, our zeta measurements in the presence of bubbles (5% H_2_O_2_) give the same s.d. as in the absence of bubbles (no H_2_O_2_), up to 6 mV (Supplementary Table 1), indicating the effectiveness of the subtraction of pressure flows due to closed capillary geometry and convective flows due to bubbles, through tracer particles. The deviations are similar to that typically obtained by standard methods.

To determine the zeta potential for each side of the colloid, we measured particle orientation changes during field direction reversals in the same set-up. Using image analysis software it was possible to directly measure the orientation of the Janus colloid as a function of time for re-alignments that proceeded via an in-plane rotation, Fig. 3e. For a spherical Janus particle with potentials *ζ*_Pt_ and *ζ*_PS_ on each half, the predicted angular velocity, 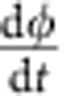, during a rotational event is a function of the angle *φ* between the normal to the dipole moment (*ζ*_Pt_−*ζ*_PS_) 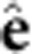 and the electric field *E*_∞_ (ref. 32):


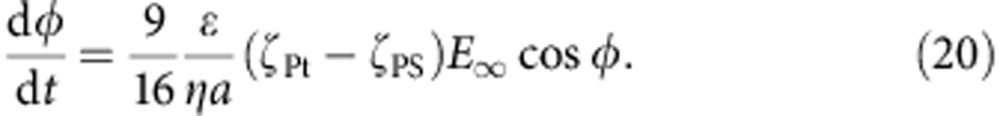


Integrating this equation with respect to *φ* and time (*t*) yields a function *f*(*φ*) that varies linearly with *t*:


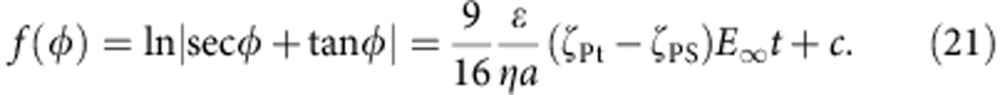


Equations (20) and (21) predict that if the particles are aligned anti-parallel to the E-field, the rotation rate will be zero and time will be ∞. This is an unstable equilibrium position. The slightest kick from the ever-present Brownian rotational motion will bring the particle to its stable equilibrium position, where the dipole is parallel to the E-field. Thus by flipping the polarity of E-field each time, the particle reestablishes its equilibrium and allows us to observe the rotation of the particle as it aligns with the E-field.

We do a frame-by-frame analysis of at least four rotations induced by field-reversal within the same field-of-view and obtain *φ*−*t* curves (Fig. 3c). Using a least-squares fit of the linear portions of corresponding versus *t* curves (Fig. 3d), we calculate the value of *ζ*_Pt_−*ζ*_PS_ for each particle observed. Together with the knowledge of *ζ*_J_=(*ζ*_Pt_+*ζ*_PS_)/2, we are able to determine *ζ*_Pt_ and *ζ*_PS_ separately (see Supplementary Table 1).

Averages and s.d. were obtained from the distribution of average zeta potential for each particle within a trial.

### Lithographic manufacture of rectangular channels

The rectangular wells used to generate the results shown in Fig. 2f were fabricated over silicon wafers in the Nanofabrication Laboratory of Materials Research Institute, Pennsylvania State University. Silicon Wafers (4″ wafer, 100′, 0.1 Ω cm conductivity and 500-μm thick) were cleaned with acetone and air-dried. The wafers were then spin-coated with 1 ml of SPR 955 photoresist (Microposit) at 900 r.p.m. for 10 s and then at 3,000 r.p.m. for 60 s. This was followed by soft-baking the coated wafers over a hot plate at 95 °C, for 60 s. The well geometry was modelled in computer-aided design and printed over a chrome-on-glass mask (Nanofabrication Laboratory, Materials Research Institute, Pennsylvania State University). For photolithography, the mask was placed in direct contact with the photoresist over the wafers. The resist was then exposed to ultraviolet radiation (intensity 8 mW cm^−2^) for 8 s in a Karl Suss MA/BA6 Contact Aligner. The exposed wafers were post-baked for 1 min over a hot plate at 95 °C to crosslink the exposed film. MF CD26 developer was used to remove the unexposed resist SPR 955 from the wafers. The mould was developed for 90 s while being agitated, followed by washing it thoroughly with DI water. After the wafers were dried with a nitrogen blower, a 30-μm-deep master pattern was created on them using deep reactive ion etching (DRIE) to yield the rectangular wells on the silicon surface. The IE was done using Alcatel Silicon DRIE in Nanofabrication Laboratory of Materials Research Institute, Pennsylvania State University and the process used was Low ARDE. After etching, the remaining resist was removed from the wafers by agitating the wafers in a solution of NanoRemover PG at 60 °C for 2 h. As a final cleaning step, the wafers were cleaned with isopropanol, hexane and acetone for 30 min each and air-dried. To homogenize the surface chemistry, the cleaned wafers were then treated with oxygen plasma (200 s.c.c.m., 400 W) for 30 min.

## Additional information

**How to cite this article:** Das, S. *et al.* Boundaries can steer active Janus spheres. *Nat. Commun.* 6:8999 doi: 10.1038/ncomms9999 (2015).

## Supplementary Material

Supplementary Information Supplementary Figures 1-2, Supplementary Tables 1-2, Supplementary Notes 1-2 and Supplementary References

Supplementary Movie 1 Radius = 0.95 μm Janus colloid settled at the bottom interface of a glass cuvette. First clip: In DI water. Free Brownian rotation and Brownian translational diffusion is observed. Second clip: in 10 % H_2_O_2_ solution. Brownian rotation about the polar axis is quenched, and directed translational propulsion away from the Pt cap is observed.

Supplementary Movie 2 Radius = 1.55 μm Janus colloid settled at the bottom interface of a glass cuvette. First clip: in DI water. Free Brownian rotation and Brownian translational diffusion is observed. Second clip: in 10 % H_2_O_2_ solution. Brownian rotation about the polar axis is quenched, and directed translational propulsion away from the Pt cap is observed.

Supplementary Movie 3 Radius = 2.4 μm Janus colloid settled at the bottom interface of a glass cuvette. First clip: in water. Brownian rotation is quenched by the mass of the cap to result in near constant bright fluorescence intensity and Brownian translational diffusion is observed. Second clip: in 10 % H_2_O_2_ solution. Brownian rotation about the polar axis is quenched, and directed translational propulsion away from the Pt cap is observed.

Supplementary Movie 4 Radius = 2.4 μm Janus colloid settled at the bottom interface of a glass cuvette in 10 % H_2_O_2_ solution, moving at the junction with a second vertical planar wall of the cuvette. Brownian rotation is quenched about two axis resulting in persistent directed motion. For the radius = 2.4 μm Janus particles , the fluorescence images also reveal that the fluorescent Janus colloids in active hemisphere is slightly canted away from the sides of the cuvette as it moves. This should be due to the increase in the gravitational torque and other non-active orientation-sensitive interactions (e.g. electrostatic, vdW, etc) that grow with size which we have ignored in the analysis in the theory in the manuscript.

Supplementary Movie 5 Two radius = 2.4 μm Janus colloids are settled at the bottom interface of a glass cuvette in 10 % H_2_O_2_ solution, moving at the junction with a second vertical planar wall of the cuvette. Brownian rotation is quenched about two axis resulting in persistent directed motion. The two colloids are moving in opposite directions and undergo a collision.

Supplementary Movie 6 Radius = 2.4 μm Janus colloid in 10 % H_2_O_2_ solution moving along the base of a 8.75 μm square cut groove lithographically manufactured in a silicon substrate
