# Supplementary material for: Boundaries can steer active Janus spheres
Source: Nat Commun. 2015 Dec 2;6:8999. doi: 10.1038/ncomms9999 (PMC4686856; doi:10.1038/ncomms9999)
Supplement: Supplementary Information — Supplementary Figures 1-2, Supplementary Tables 1-2, Supplementary Notes 1-2 and Supplementary References [file ncomms9999-s1.pdf]

Supplementary Information

Supplementary Figures:

Supplementary Figure 1: Relationship between particle size, fuel concentration and velocity

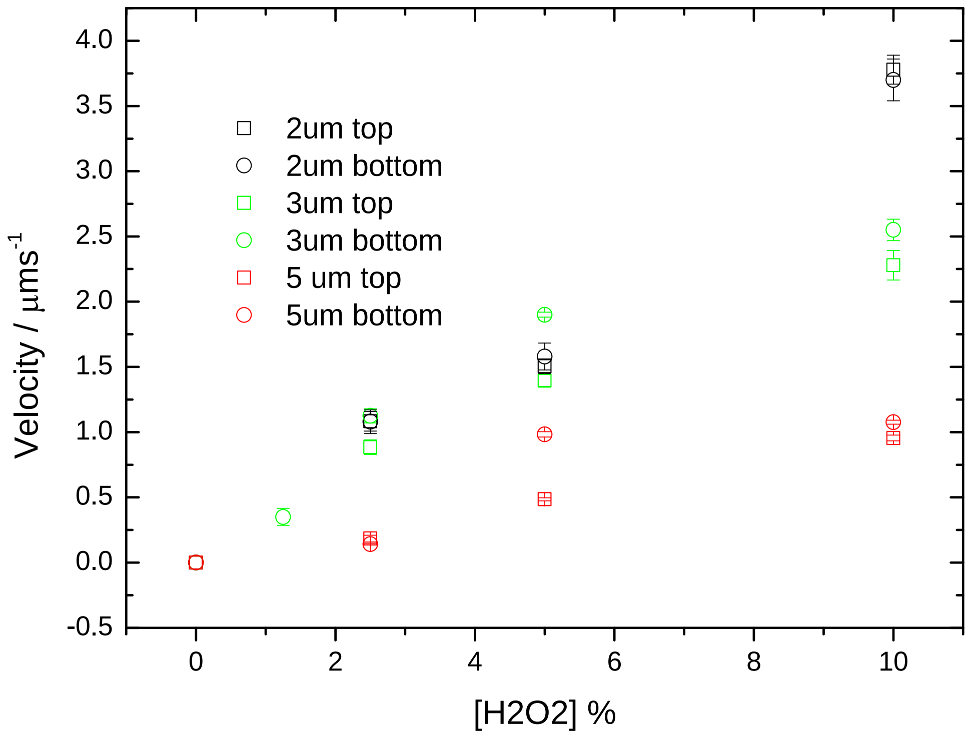

Supplementary Figure 1: **Average propulsion velocity versus fuel concentration.** Mean velocity as a function of hydrogen peroxide (H_2O_2) concentration for all of the Pt-PS (Platinum-Polystyrene) Janus particles investigated in section 1. “Top” refers to particles swimming at the top surface of the cuvette, and “Bottom” to particles sediment at the bottom of the cuvette.

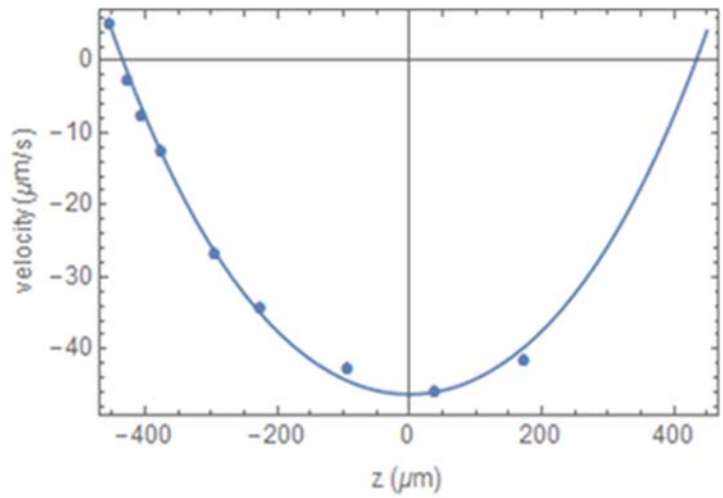

(a)

Sample	ζ (mV)
ζ_{spsl}, C1	-102.6 ± 1.7
ζ_{spsl}, C2	-100.7 ± 1.6
ζ_{w}, C1	-124.0 ± 2.9
ζ_{w}, C2	-125.4 ± 2.4
ζ_{spsl}, Zetasizer	-106.5 ± 4.8

(b)

Supplementary Figure 2: **Zeta Potential Calibration Details**. (a) Markers represent experimentally measured velocities of sPSL particles as a function of height from the centre. The solid line represents the best fit parabola using ζ_{spsl} and ζ_{wall} as parameters in Bowen's equation. (b) Shows the ζ_{spsl} and ζ_{wall} calculated from the fit in fig a for 2 independent trials, C1 and C2. The last row shows the ζ_{spsl} measured using Malvern Zetasizer.

Supplementary Tables:

Supplementary Table 1

a (μm)	D_∞ (μm^2 s^{-1})	$D_{//}$ (μm^2 s^{-1})		h (μm)	
		Water	$I = 8 \times 10^{-5}$ M	Water	$I = 8 \times 10^{-5}$ M
0.95	0.232	0.163 (0.039)	0.082 (0.026)	0.860	0.030
1.55	0.142	0.095 (0.018)	0.046 (0.011)	1.068	0.040
2.40	0.092	0.042 (0.013)	0.039 (0.014)	0.379	0.240

Supplementary Table 1: Diffusion coefficients parallel to a wall ($D_{||}$) and the corresponding gap heights (h) of a suspension of Janus colloids in both water and KNO_3 solution at 21°C. Values in brackets are the standard deviation of the mean values. A suspension of colloidal particles diffusing well away from a wall has a Brownian diffusion coefficient D_∞ described by the Stokes-Einstein relation ($D_\infty = k_B T / 6\pi\eta a$). As the colloids settle under gravity and reach an equilibrium height above the bottom surface of their container, their diffusion is hindered by an additional hydrodynamic drag. We can use these deviations from the bulk diffusion coefficient for colloids moving near a wall to estimate the colloidal suspension height above a wall, as described by Kazoe.[1] The hindrance factor for diffusion parallel to a surface is given by Supplementary Equation. 1.

$$\frac{D_{||}}{D_\infty} = 1 - \frac{9}{16}\left(\frac{a}{z}\right) + \frac{1}{8}\left(\frac{a}{z}\right)^3 - \frac{45}{256}\left(\frac{a}{z}\right)^4 - \frac{1}{16}\left(\frac{a}{z}\right)^5 \qquad (1)$$

where $D_{||}$ is the diffusion coefficient parallel to the surface and the gap height h is contained in the $z = a + h$ term.[2,3]

To estimate the height of the particles above a fused quartz silica wall we suspended a mixture of green fluorescent Janus colloids (a = 0.95, 1.55 and 2.40 μm) in water (Elga Purelab Option, 15 MΩ cm) and allowed them to settle out. With the Janus colloids at their equilibrium height above the bottom wall of the cell, videos of 25 individual particles of each size were recorded at a frame rate of 100 Hz (Andor Neo camera). Image analysis algorithms were used to extract 2D trajectories of the particles diffusing in the (x, y) plane and the mean squared displacements ΔL^2 computed. A fit

to ΔL^2 yielded the hindered diffusion coefficient $D_{||}$. As it is problematic to extract accurate values for Brownian diffusion coefficients for Janus colloids also undergoing significant propulsion, we use measurements on unfuelled colloids as a proxy for the active colloid parameters. The strongest H_2O_2 solution (Sigma Aldrich, 30.0 wt%, pKa = 11.75) that we add to a suspension of Janus sphere swimmer particles to observe swimming behaviour contains the stabiliser dipicolinic acid at a concentration of 40 mg L^{-1} (pKa = 2.2) giving an estimated ionic strength, $I = 8 \times 10^{-5}$ M, at 10 % wt. To allow for the expected screening effect in this solution we used a KNO_3 solution with the same ionic strength. These results, together with those in pure water are shown.

Supplementary Table 2

ζ_J	σ_ζ	$\zeta_{Pt} - \zeta_{PS}$	σ_ζ	ζ_{Pt}	σ_ζ	ζ_{PS}	σ_ζ
5 % H_2O_2, t = 10 mins							
-83.1	5.0	38.4	2.6	-63.9	5.2	-102.3	5.2
-79.5	5.5	38.5	2.2	-60.3	5.6	-98.8	5.6
-93.9	5.3	32.2	5.9	-77.8	6.0	-110.0	6.0
-90.1	5.4	36.3	6.4	-71.9	6.3	-108.3	6.3
-84.2	5.3	42.9	5.5	-62.7	6.0	-105.6	6.0
Averages							
-86.2	5.3	37.7	4.5	-67.3	5.8	-105.0	5.8
5 % H_2O_2, t = 60 mins							
-99.5	5.8	15.9	4.6	-91.5	6.3	-107.4	6.3
-98.4	4.9	22.8	2.8	-87.0	5.1	-109.8	5.1
-97.8	5.2	20.3	6.5	-87.7	6.1	-108.0	6.1
-98.3	6.1	27.2	5.7	-84.7	6.7	-111.9	6.7
-95.0	5.3						
Averages							
-97.8	5.4	21.5	4.9	-87.7	6.0	-109.3	6.0
Water, t = 10 mins							
-89.1	4.6	14.2	2.1	-82.0	4.7	-96.2	4.7
-87.1	6.6	20.4	6.9	-76.9	7.4	-97.2	7.4
-89.1	5.6	12.5	1.2	-82.8	5.7	-95.3	5.7
-92.4	4.8	10.6	1.6	-87.2	4.9	-97.7	4.9
-90.1	6.3						
Averages							
-89.5	5.6	14.4	2.9	-82.2	5.7	-96.6	5.7
Water, t = 60 mins							
-88.2	4.4	14.6	4.0	-80.9	4.8	-95.5	4.8
-87.7	5.2	13.7	2.5	-80.9	5.3	-94.6	5.3
-89.7	5.3	16.3	2.4	-81.5	5.4	-97.8	5.4
-86.3	5.3	14.4	2.7	-79.1	5.5	-93.5	5.5
-85.3	4.6	13.9	0.4	-78.3	4.6	-92.2	4.6
Averages							
-87.4	4.9	14.6	2.4	-80.1	5.1	-94.7	5.1

Supplementary Table 2: Measured values of zeta potential of the Janus particle, platinum and polystyrene halves of the Janus particle in water and 5% H_2O_2 at time 10 mins and 60 mins. This table shows the actual values of the zeta potential measured for each particle and the averages for each trial, referenced in Figure 3g of the manuscript. The averages indicate very good consistency within a batch and the intra-batch variability is similar for trials with and without H_2O_2. Thus it is

clear that the method of subtracting velocities of tracer particles in the presence of bubbles is effective in removing the effect of complex convective flows around a bubble.

Supplementary Notes:

Supplementary Note 1: Sample Preparation and Gravitaxis of Janus Colloids

When Janus colloid solutions are evenly dispersed into a low volume rectangular section glass cuvette (Suprasil glass cuvettes from Hellma Analytics) containing water, sedimentation leads to accumulation at the bottom planar surface. However, in the presence of hydrogen peroxide fuel, a propulsion velocity due to asymmetrical catalytic fuel decomposition is introduced. With fuel, radius=1 μm Janus colloids particles now rapidly arrive at both the top and bottom interior planar surfaces of the cuvette; whereas for radius=1.55/2.4 μm colloids, accumulation preferentially occurs at the top interface. This biased accumulation for the larger colloids is due to the asymmetrical mass distribution caused by the dense platinum hemisphere resulting in gravitaxis.[4] For all the sizes of colloids measured, microscopic examination of the Janus particles at the interior cuvette surfaces, revealed persistent 2D enhanced in plane motion at the interfaces. Furthermore, if the cuvettes are inverted, the enhanced in plane motion continued, despite the reversal in gravitational field direction. Consequently observations for many catalytic PS-Pt Janus colloids moving in 2D along planar interfaces could be made in two gravitational orientations (+g at the top surface, and –g for the bottom, Figure 1d of the manuscript). It is interesting to note that inactive colloids sediment away from the top surface, whereas the active fuelled colloids investigated here do not, and remain at the top interface. No significant changes in the surface-colloid interactions have been found on the addition of fuel to explain this effect. Instead a small upwards component of propulsion sufficient for the colloid to maintain contact with the wall and prevent sedimentation back into the bulk solution may be present. A force balance approach can be applied to this scenario, and shows that e.g. for a=1 μm colloid, a modest cant angle towards the top surface (ϴ=86°) is sufficient to balance sedimentation. These small incline angles will be difficult to detect within our experimental ability to resolve ϴ in this geometry, however cant angles deviating from 90 ° were observed for confinement in multi-planar geometries (See Supplementary Movie 4). This analysis suggests that

reducing propulsion velocity and increasing particle size will require a greater cant angle to overcome sedimentation, and that also for sufficiently large colloids, sedimentation will become inevitable, as due to the (1/a) dependency on propulsion velocity there will not be sufficient force to overcome sedimentation irrespective of Θ. To illustrate this experimentally, we investigated some a=5 μm colloids, and found that for low fuel concentrations (approximately 1.5 %), sedimentation from the top surface was observed.

Supplementary Note 2: Exploring the limits for boundary steering phenomena

For boundary steering to be exploited for applications, it is important that active colloids retain the ability to be directed or confined at boundaries for sufficient durations to enable transport over useful distances. Encouragingly in this context, during our experiments, which involved repeated observations of hundreds of randomly selected particles for periods of several minutes, we observed very few cases where a particle would detach from a planar interface, or cease to be steered by a multi-planar geometry. Indeed we have shown that as a result micron sized active colloids can be steered by boundaries over macroscopic cm length scales. Because of this persistence of motion it is experimentally difficult to investigate the frequency of "detachment" events where active colloids remove from a boundary or a plane. However, by inspection it appears that the residence time for a given active colloid will depend on the degree of rotational confinement, and the consequences of temporary misalignment. Our theoretical analysis and experimental data has indicated that at slower propulsion speeds rotational quenching is diminished, and so we can expect a higher frequency of events where the active colloid is no longer completely orientated parallel to the nearby interfaces. Therefore we may predict lower residence times for slower moving particles. However, it is also clear that momentary misalignment may not necessarily lead to a particle separating from the interface. In some cases the misalignment may cause the propulsion vector to point towards the solid interface, which may not lead to detachment. Even if the propulsion vector is orientated away from the steering/confining interfaces, this may not generate sufficient force to

result in the active colloid returning to “bulk”. This specifics of this case will depend on the geometry and size of the colloid, for example, for a colloid to leave the lower planar surface of a container will require it to produce sufficient upwards propulsion velocity to overcome its tendency to sediment, a factor that will actually encourage low velocity colloids to remain at the surface despite more frequent misalignments. Another consideration is stochastic variations in distance of the colloid from the constraining interface. Our theoretical analysis shows that the pre-factor determining the strength of orientational quenching has an inverse cubic relationship with separation from the wall. This leads to the possibility of Brownian “kicks” randomly translating the active colloid sufficiently far from the interface that orientational quenching is no longer experienced. Our experiments confirm that these events do not happen frequently at the sizes we have investigated. However, this effect could become limiting at smaller colloidal sizes, as Brownian translational diffusion rate increases.

While this general discussion applies to all the geometries considered, we also present a more detailed consideration of the differences between the single plane active colloid arrangement which we subjected to rigorous analysis, and the multi-plane arrangements. It was our hypothesis that adding an additional plane or planes beyond the single plane system would display the same hydrodynamic confinement phenomena, however now with additional axis of rotation being quenched, resulting in boundary steering. In comparison to the single planar system, the arguments we developed for hydrodynamic rotational confinement being a dominant factor in the system remain valid: the substrate material was unchanged for experiments conducted at an “edge”, and silicon substrates were used for the groove experiments which have also similar properties. Consequently the electrostatic interactions are expected to stay the same. We observed that all sized particles moving within ten percent hydrogen peroxide solutions experienced prolonged boundary steering with long residence times at the edge formed between either the upper or lower planar cuvette surface and the vertical cuvette sidewall. This steering effect also tolerated the moderate curvature of the steering boundary found at the corners of the cuvette. As reported for

the single plane case, the fluorescence intensity of the active colloid remained unchanged during confinement, and a constant "phase of the moon" was observed following the boundary direction and correlating with the expected propulsion direction away from the platinum cap. This shows that the boundary steering did correlate with the active colloid orientation confinement relative to both planes. For the case of the grooved lithographic example, the additional constraint that the colloid could physically fit within the groove was imposed, which in practise resulted in active colloids of a given size entering grooves that were approximately 2 µm wider than their diameters. Due to optical reflections within the groove, it was not possible to determine the orientation of the active colloid as it followed a groove, however persistent propulsion motion along the groove was observed until the colloid reached the end at which point it became stuck. At this point microscopy did allow the orientation of the colloid to be viewed, and this was found to be aligned as expected relative to both boundaries. As mentioned in the main text, the groove system possesses the potential to prevent Brownian kicks from allowing smaller colloids to escape orientational confinement, which could occur if only 2 planes are used to steer motion.

Supplementary References

1. Kazoe, Y. & Yoda, M. Measurements of the near-wall hindered diffusion of colloidal particles in the presence of an electric field. *Appl. Phys. Lett.* **99,** 2009–2012 (2011).

2. Goldman, a. J., Cox, R. G. & Brenner, H. Slow viscous motion of a sphere parallel to a plane wall—II Couette flow. *Chem. Eng. Sci.* **22,** 653–660 (1967).

3. Banerjee, A. & Kihm, K. Experimental verification of near-wall hindered diffusion for the Brownian motion of nanoparticles using evanescent wave microscopy. *Phys. Rev. E* **72,** 042101 (2005).

4. Campbell, A. I. & Ebbens, S. J. Gravitaxis in spherical janus swimming devices. *Langmuir* **29,** 14066–73 (2013).
